# The influence of indigenous documentary narratives on cultural resilience and mental health awareness

**DOI:** 10.3389/fpsyg.2026.1975220

**Published:** 2026-10-14

**Authors:** Pengcheng Li, Chenjin Jia, Yanhao Chen

**Affiliations:** 1 School of Communication, Universiti Sains Malaysia, Penang, Malaysia; 2 School of Literature and Journalism Media, Yibin University, Yibin, Sichuan, China

**Keywords:** cultural authenticity, cultural identification, cultural resilience, help-seeking attitudes, indigenous communities, indigenous documentary narratives, mental health awareness, mental health stigma

## Abstract

Indigenous documentary narratives may provide culturally grounded contexts for understanding identity, resilience and mental health, yet empirical evidence linking narrative engagement with these interconnected outcomes remains limited, particularly within Indigenous communities in China. This cross-sectional mixed-methods observational study examined associations among narrative engagement, perceived cultural authenticity, cultural identification, cultural resilience, mental health awareness, stigma and help-seeking attitudes among 480 adults from ethnic minority communities in Yunnan and Hunan Provinces, China, with 36 participants contributing qualitative data. After a standardized viewing session involving four documentaries representing Yi, Miao, Bai and Tujia/Miao narratives, participants completed culturally adapted measures of narrative engagement and cultural and mental-health constructs. Confirmatory factor analysis supported the final 60-item, six-factor measurement model (*χ*^2^(1,695) = 3,284.15, *χ*^2^/df = 1.937, CFI = 0.941, TLI = 0.938, RMSEA = 0.044, 95% CI [0.042, 0.046], SRMR = 0.048), with satisfactory reliability and convergent validity. Narrative engagement was positively associated with cultural resilience (*r* = 0.51), mental health awareness (*r* = 0.44), and help-seeking attitudes, while showing an inverse association with mental health stigma (*r* = −0.35; all *p* < 0.001). Multivariable models explained 53.4% of the variance in cultural resilience and 34.8% of the variance in mental health awareness. Covariate-adjusted statistical indirect associations were observed through cultural identification (*β* = 0.225, 95% BCa CI [0.168, 0.291]) and perceived cultural authenticity (*β* = 0.114, 95% CI [0.058, 0.144]). Qualitative analysis identified cultural mirroring, normalization of distress through cultural metaphors, and intergenerational dialogue as central themes. Overall, the findings identify interconnected statistical associations among narrative, cultural and mental-health variables, but do not establish temporal or causal pathways. Longitudinal and comparative studies are needed to examine the temporal stability and potential mechanisms underlying these associations.

## 1 Introduction

Indigenous communities possess rich systems of knowledge, cultural memory, collective identity and relational practices that have been transmitted across generations through oral histories, ceremonies, visual traditions, community practices and place-based narratives (Kennedy and Silverstein, 2023). These cultural systems have frequently been represented through external perspectives that simplify Indigenous experiences, marginalize community voices, or frame Indigenous populations primarily through narratives of disadvantage. Representational inequalities have important psychological and social consequences because cultural identity, historical memory, community belonging and perceived continuity are closely interconnected with individual and collective well-being. In this context, documentary media produced with, by, or about Indigenous communities has increasingly emerged as a potentially important mechanism for cultural representation and psychosocial engagement (Azocar and Carstarphen, 2026). Indigenous documentary narratives can preserve endangered cultural knowledge while simultaneously creating spaces in which communities articulate their own histories, identities, challenges, aspirations and interpretations of well-being. From a psychological perspective, the relevance of documentary narratives depends not only on whether cultural information is represented, but also on how viewers attend to, emotionally engage with, recognize and interpret that representation. Narrative engagement may therefore provide an important link between culturally grounded media content and psychosocial outcomes, particularly when narratives are perceived as authentic and culturally recognizable. The psychological significance of these narratives therefore extends beyond cultural preservation to questions of resilience, identity development, social connectedness and mental health awareness.

Cultural resilience refers to the ability to sustain, reconstruct and adapt cultural identity and collective functioning in the face of historical disruption, discrimination, displacement, socioeconomic disadvantage and other structural adversity. While resilience is not the same as individual endurance against adversity, it is rooted in relationships, cultural practices, institutions, collective memory and access to community resources (Sauda, 2025a). When younger generations are able to experience ancestral knowledge, language, traditions, land relationships and community histories, the cultural resilience of an Indigenous community may be supported through processes of cultural recognition, identity continuity and intergenerational connection (Nogueira, 2025). Documentary narratives can provide one context in which these processes become visible and interpretable, transforming culturally significant experiences into audiovisual records that can circulate within and outside of communities through storytelling, archives, Elder interviews, community-based testimonies, artistic expression and representations of everyday cultural practices. Perceived cultural authenticity may further strengthen the relevance of the narrative by signaling that cultural experiences are being represented in ways that correspond to community meanings rather than solely to external interpretations.

Documentary narratives can also be understood as contexts in which psychological processes relevant to resilience may be activated or represented and the mechanisms through which documentary engagement is associated with resilience are of particular interest to psychology. Narrative exposure can contribute to how individuals conceptualize their selves and communities and documentary narratives can offer interpretive frameworks for experiences of historical adversity and cultural survival (Folorunsho, 2026). Narrative transportation, including sustained attentional absorption and emotional involvement with a story, may make culturally meaningful experiences more psychologically salient and provide viewers with opportunities to reflect on shared identity and collective experience. Representations of Indigenous identities, languages, traditions and lived experiences may be associated with stronger cultural identification and perceptions of collective efficacy when viewers recognize aspects of themselves and their communities in documentary narratives. Inaccurate or stereotypical representations may reinforce stigma, cultural alienation, or psychological distress. Documentary storytelling can be valuable to Indigenous communities, but visibility is not enough; authenticity, community participation, narrative ownership and ethical representation are also key (Ilori et al., 2025). Participatory documentary approaches place community members as holders of knowledge and narrative agents rather than as passive subjects of observation, making community participation and narrative ownership particularly relevant to the interpretation of authenticity and cultural identification. These considerations suggest that engagement with Indigenous documentary narratives should be examined as multidimensional experience involving attention, emotional involvement, perceived authenticity and cultural identification rather than as simple exposure to media content.

The potential role of Indigenous documentary narratives in addressing mental health is a related but less studied dimension. Individual, familial, cultural, historical, socioeconomic and structural determinants all play a role in mental health challenges in Indigenous populations (Kirkbride et al., 2024). Clinical language or communication models that do not align with local cultural meanings may limit traditional approaches to mental health communication. Documentary narratives can be a bridge between professional discourse and lived experience, making mental health concepts more comprehensible and socially relevant and bringing the distance between professional discourse and lived experience (Yeo et al., 2022). Documentaries that feature personal testimonies and community narratives can encourage dialogue and help raise awareness of mental health issues by putting psychological experiences into the context of family, community, culture, history and relationship to land. This process may be particularly relevant when psychological experiences are communicated through culturally familiar stories rather than through exclusively clinical terminology. Culturally grounded narratives can provide examples of how distress is recognized, discussed and managed within families and communities, potentially supporting social learning by making coping responses and help-seeking behaviors more observable and discussable.

Storytelling can also be relevant to mental health awareness and stigma through processes of normalization and social interpretation, particularly where mental health difficulties may be associated with silence, shame, fear of judgment, or lack of confidence in formal services (Syed and McLean, 2022). Culturally grounded narratives can create opportunities to interpret emotional distress within familiar cultural and relational contexts, thereby making conversations about psychological well-being more socially recognizable. The presentation of characters or community members discussing distress may also provide forms of social learning in which viewers observe how psychological experiences are named, understood, communicated and responded to by others. Indigenous documentary narratives may also highlight culturally meaningful protective factors including family cohesion, traditional practices, spirituality, language revitalization, connection with ancestral lands, collective activities and intergenerational relationships, thereby providing culturally situated examples of resources that may be relevant to resilience and well-being. Intergenerational dialogue is another potentially important mechanism: documentary narratives can provide a shared cultural reference point through which younger and older community members discuss changing traditions, historical experiences, emotional difficulties and contemporary mental-health concerns. These processes may shift attention from deficit-based understandings to strengths-oriented perspectives that see Indigenous communities as sources of cultural resources to support well-being.

Empirical evidence examining the engagement with Indigenous documentary narratives is associated with psychological outcomes, as much of the existing research has focused on Indigenous media representation, cultural revitalization, storytelling, or mental health separately, without considering the integration of these dimensions, including whether narrative engagement, perceived cultural authenticity and cultural identification are associated with measurable differences in cultural resilience, mental health awareness, help-seeking attitudes and mental health stigma (Ajani et al., 2024). Limited empirical work has examined whether cultural identification and perceived authenticity statistically account for part of the association between narrative engagement and cultural or mental-health outcomes. There is also limited understanding of how community members themselves describe the processes of cultural mirroring, normalization of distress, social learning and intergenerational dialogue that may accompany engagement with culturally grounded narratives. These gaps are critical for developing evidence-based approaches to culturally responsive mental health communication.

The present cross-sectional mixed-methods study examines associations among Indigenous documentary narrative engagement, perceived cultural authenticity, cultural identification, cultural resilience, mental health awareness, mental health stigma and help-seeking attitudes among adults from ethnic minority communities in Yunnan and Hunan Provinces, China. Rather than testing pre–post change or the causal effect of documentary viewing, the study examines how these variables are related following a standardized documentary-viewing session. The study evaluates greater narrative engagement associated with stronger cultural identification and cultural resilience, greater mental health awareness, lower mental health stigma and more favorable help-seeking attitudes. It further examines the cultural identification and perceived cultural authenticity statistically account for part of the observed associations between narrative engagement and cultural resilience or mental health awareness. The qualitative component uses purposive sampling to explore participants’ interpretations of cultural representation, emotional engagement, normalization of distress and intergenerational dialogue, thereby providing contextual insight into the quantitative associations. By integrating perspectives from cultural psychology, narrative psychology, media psychology and culturally responsive mental health communication, the study seeks to clarify theoretically relevant associations between documentary engagement, cultural processes and mental-health-related constructs. The findings are intended to inform future longitudinal, experimental and Indigenous-led research rather than establish temporal or causal effects of documentary narratives.

## 2 Materials and methods

### 2.1 Study design and conceptual framework

A cross-sectional mixed-methods observational design was used to examine associations among Indigenous documentary narrative engagement, perceived cultural authenticity, cultural identification, cultural resilience, mental health awareness, mental health stigma and help-seeking attitudes, while also exploring participants’ interpretations of culturally grounded narratives (Hendayani et al., 2026). Participants completed a standardized documentary-viewing session and subsequently completed the study measures, allowing relationships among narrative engagement, cultural variables and mental-health outcomes to be examined within the same assessment period. The quantitative analyses estimated cross-sectional associations and statistical indirect associations rather than causal effects, intervention effects or longitudinal change. The qualitative component consisted of semi-structured interviews with a purposively selected subgroup of participants following completion of the quantitative assessment (Kohrman et al., 2024).

The conceptual model drew from cultural resilience theory, narrative psychology, social identity perspectives and culturally responsive mental health communication. Culturally authentic documentary narratives were conceptualized as potentially relevant to cultural identification, perceived cultural continuity and cultural resilience, whereas narratives addressing emotional well-being, psychological distress, stigma, recovery and culturally meaningful support were conceptualized as potentially relevant to mental health awareness and help-seeking attitudes. Narrative engagement, perceived cultural authenticity and cultural identification were examined as theoretically relevant explanatory variables rather than confirmed causal mediators. The framework incorporated narrative transportation, attentional absorption, emotional involvement, cultural mirroring, social learning, normalization of distress and intergenerational dialogue as potential processes through which documentary engagement may be meaningfully related to psychosocial outcomes. Age, gender, education, cultural participation, previous Indigenous-media exposure and geographic/community setting were considered as contextual characteristics. The mixed-methods design allowed quantitative associations to be interpreted alongside participants’ accounts of cultural representation, identity, emotional responses, intergenerational learning and mental-health communication.

### 2.2 Participants, sampling, recruitment and sociodemographic characteristics

Participants were recruited through community organizations, Indigenous cultural centers, educational institutions, community networks and Indigenous-led communication channels within the predetermined study setting. Recruitment procedures were developed in consultation with community representatives to minimize coercion and ensure that participation was accessible and culturally appropriate. Eligibility criteria were age ≥18 years, self-identification as Indigenous or recognized membership within the participating community (Wilson, 2020), ability to view and understand the documentary material and provision of written informed consent. Participants were excluded if they were unable to complete the viewing protocol or outcome assessments, had previously participated in the development or evaluation of the selected documentary material, or had a communication limitation that prevented completion of the study instruments despite reasonable accommodations.

Purposive and community-based sampling was employed to obtain variation in age, gender, educational attainment, geographic setting, cultural participation and previous exposure to Indigenous media (Yuan and Ren, 2025). Recruitment occurred from September 2024 to April 2025. A total of 572 individuals were screened; 42 were excluded before enrollment, 530 provided consent, 32 discontinued before completion, 498 completed the quantitative procedures and 18 were excluded during prespecified data-quality screening, resulting in a final quantitative sample of 480 participants. A purposive qualitative subsample of 36 participants subsequently participated in semi-structured interviews. *A priori* sample-size estimation was performed using G*Power assuming a small effect size of *f*^2^ = 0.04, *α* = 0.05, statistical power = 0.90 and 12 planned predictors/covariates, yielding a minimum required sample of 412 participants (Shook-Sa and Hudgens, 2022). The final sample therefore exceeded the prespecified requirement. Sociodemographic and contextual variables included age, gender, education, geographic/community setting, cultural participation, previous Indigenous-media exposure and relevant cultural-context characteristics. These variables were collected because demographic and cultural contexts may influence interpretation of documentary narratives and may also be associated with psychosocial outcomes. Participant flow was documented from screening through quantitative completion and qualitative participation.

### 2.3 Documentary narrative selection, cultural governance and exposure protocol

A content-selection procedure was applied to identify documentary narratives suitable for the standardized viewing session (Falcone, 2024). Documentary narratives were eligible if they centered Indigenous individuals, families, communities, cultural practices, histories, languages, knowledge systems or contemporary experiences and provided substantial representation of Indigenous perspectives (Jiang et al., 2025). Productions were selected according to predefined domains including cultural identity, language, traditional knowledge, historical experience, relationships with land and community, intergenerational transmission, cultural continuity, resilience, emotional well-being, mental health, stigma, recovery and community support (Falcone, 2024). Priority was given to productions involving Indigenous filmmakers, Indigenous production organizations or meaningful Indigenous participation in research, production, narration or editorial decision-making.

The final viewing set comprised four culturally grounded documentaries representing Yi, Miao, Bai and Tujia/Miao narratives: *Echoes of the Torch* (Yi; 2022; 38 min), *Silver and Soil* (Miao; 2021; 42 min), *Wind and Water Courtyards* (Bai; 2023; 28 min) and *Voices Across the River* (Tujia/Miao; 2020; 32 min). Documentary selection included community review to identify material that was culturally respectful, contextually appropriate and unlikely to reproduce harmful stereotypes (Wierucka, 2024). Material containing gratuitous stereotyping, sensationalized trauma, exploitative imagery, unauthorized disclosure of culturally restricted knowledge or decontextualized representations was excluded (Jiang et al., 2025). Documentary metadata were recorded by title, production year, region, duration, language, narrative focus, Indigenous participation and cultural domains represented. Where production or access information was not publicly distributable because of copyright or community-governance considerations, the study retained documentary identification and access records without reproducing restricted material. Participants viewed the assigned documentary under standardized conditions, using the same presentation format without interruption. Instructions emphasized attentive viewing without providing interpretive guidance. Immediately after viewing, participants completed the study measures. Viewing completion, attentional engagement, emotional involvement, narrative transportation, perceived relevance and cultural authenticity were assessed as characteristics of documentary engagement rather than evidence of an intervention effect (Fuller, 2022).

### 2.4 Measurement of cultural identity, cultural resilience and narrative engagement

A dedicated measurement framework was used to provide explicit information on the instruments, item content, response formats, scoring and cultural adaptation procedures. Cultural identity was conceptualized as distinct from, although related to, cultural resilience. Cultural resilience referred to the perceived capacity to sustain cultural continuity, adapt to adversity, maintain culturally meaningful practices and transmit knowledge across generations (Zhou et al., 2025). Psychological resilience was conceptually distinguished from cultural resilience to avoid equating cultural strength with absence of psychological difficulty (Yang and Gao, 2025).

Cultural identification was assessed using a six-item adapted version of the Multigroup Ethnic Identity Measure–Revised, with items addressing cultural/ethnic exploration and commitment. Responses were recorded on a five-point scale, with higher scores indicating stronger cultural identification. Cultural resilience was assessed using a 14-item adapted Native Reliance and Resilience measure addressing culturally grounded continuity, community reliance and resilience-related experiences. Items were scored using the study-specific agreement format, with higher scores indicating stronger perceived cultural resilience. Additional contextual items assessed cultural continuity, intergenerational knowledge transmission, participation in cultural practices, community belonging, collective efficacy, connection with culturally significant places and perceived importance of Indigenous language and traditions. These additional items were examined separately unless incorporated into a prespecified composite.

Narrative engagement was assessed using a 12-item adapted narrative transportation measure based on attentional absorption, cognitive involvement, imagery and emotional engagement with the narrative. Responses used a seven-point scale, with higher scores indicating greater engagement. Attentional absorption referred to sustained cognitive attention to the documentary, whereas emotional involvement referred to the intensity of affective engagement. Perceived cultural authenticity was assessed using an eight-item adapted measure addressing accurate and respectful representation of Indigenous people, practices, histories and lived experiences, with responses recorded on a seven-point agreement scale. Adaptation involved forward translation into Mandarin, back-translation, review by a five-member expert panel, community Elder consultation and pilot testing with 30 participants. Reliability was evaluated using Cronbach’s α and McDonald’s ω (Madadizadeh and Bahariniya, 2025), while CFA was used to examine the measurement structure and convergent/discriminant validity.

### 2.5 Assessment of mental health awareness, stigma, help-seeking and service accessibility

Mental health awareness was operationalized as knowledge and recognition of psychological well-being and mental-health difficulties, awareness of available sources of support, understanding of circumstances in which professional or community assistance may be appropriate and confidence in discussing emotional well-being. Mental health awareness was differentiated from psychological symptom severity and was not treated as a diagnostic construct (Gu et al., 2022). The study used a 12-item culturally adapted mental-health-awareness measure derived from the specified mental-health-literacy framework, with items addressing recognition of mental-health concepts, awareness of support options and confidence in discussing mental-health concerns. Responses were scored using the study-specific response anchors, with higher scores indicating greater mental health awareness.

Mental health stigma was assessed using a 10-item culturally adapted measure covering negative stereotypes, shame, social distancing and attitudes toward individuals experiencing psychological difficulties (Perry et al., 2022). Higher scores represented greater stigma. Help-seeking attitudes were assessed using a 10-item adapted measure addressing willingness to discuss emotional problems, openness to professional psychological services, acceptance of community-based support, perceived usefulness of psychological services and confidence in seeking assistance (Kamal and Sánchez-Peña, 2026). Instrument wording was reviewed for cultural clarity and potentially stigmatizing or pathologizing terminology was minimized.

Participants additionally reported perceived accessibility, affordability, cultural responsiveness, confidentiality and trustworthiness of available mental-health services. Because Indigenous communities may use culturally grounded approaches to emotional and psychological well-being, traditional healing, community support, spirituality, cultural practices and intergenerational guidance were not treated as inherently incompatible with professional psychological care (Williams and Sikrweqe, 2026). Where relevant, participants were asked whether integration of culturally meaningful practices with formal mental-health services was acceptable. Reliability and construct validity were evaluated using the same general procedures applied to the cultural measures (Aghababaeian et al., 2024). All mental-health variables were analyzed as cross-sectional constructs. No pre–post change scores, intervention effects, or claims of reduced stigma or improved awareness following documentary exposure were estimated. This distinction ensured that mental-health findings remained consistent with the observational design.

### 2.6 Narrative engagement, statistical indirect associations and moderating factors

Narrative engagement was assessed to characterize the depth and quality of participants’ psychological involvement with the documentary narratives (Wang, 2026; Xiong et al., 2026). Domains included attentional absorption, emotional involvement, narrative transportation, identification with represented individuals, perceived relevance, perceived authenticity, cultural resonance and perceived respectfulness of representation. Participants rated the extent to which the documentary captured community perspectives accurately, reflected culturally meaningful experiences, encouraged reflection on personal or collective identity and stimulated discussion of psychological well-being. Perceived cultural authenticity was assessed using an adapted 8-item measure of cultural authenticity. Items assessed the extent to which participants perceived the documentary narratives as culturally credible, representative, contextually grounded and consistent with lived community experiences. Responses were recorded on 7-point Likert scale ranging from 1 (strongly disagree) to 7 (strongly agree). The measure was forward-translated into mandarin and independently back-translated, followed by review by the study’s expert panel and community representatives. A pilot assessment with 30 participants was used to evaluate comprehensibility and cultural appropriateness before the main study. Higher scores indicated greater perceived cultural authenticity (Caffer et al., 2024). Narrative transportation reflected cognitive and emotional immersion in the story.

The analysis distinguished simple documentary viewing from meaningful narrative engagement. Attentional absorption referred to sustained attention and cognitive involvement; emotional involvement referred to affective engagement; cultural mirroring referred to recognition of familiar cultural experiences, symbols, language or community histories; social learning referred to observing culturally meaningful ways of understanding or responding to psychological experiences; normalization referred to the presentation of emotional distress within familiar social and cultural contexts; and intergenerational dialogue referred to discussion of cultural and mental-health meanings across generations. These constructs were treated as theoretically informed explanatory variables rather than established causal mechanisms.

Cultural identification was examined as a statistical indirect-association variable for cultural resilience, whereas perceived cultural authenticity was examined as a statistical indirect-association variable for mental health awareness. The indirect effects were estimated using 5,000 non-parametric bootstrap resamples because products of regression coefficients may have asymmetric sampling distributions. Bootstrap confidence intervals therefore provided inference without requiring normality of the indirect effect. Age, gender, educational attainment, cultural participation, previous Indigenous-media exposure, geographic/community setting and mental-health awareness were considered as potential contextual moderators or covariates based on their theoretical relevance to narrative interpretation and psychosocial outcomes. Continuous predictors were centered before interaction terms were formed where moderation was examined. Mediators and moderators were therefore kept conceptually distinct throughout the analysis.

### 2.7 Qualitative interviews, reflexivity and thematic analysis

A purposive subsample of participants was selected for semi-structured interviews to provide contextual interpretation of the quantitative findings. Purposive sampling was selected because the qualitative component sought information-rich and diverse accounts rather than statistical representativeness. Selection sought maximum variation across age, gender, cultural engagement, documentary response and representation of the major cultural and geographic contexts included in the quantitative sample (Dahal et al., 2024). Thirty-six participants completed interviews lasting 35–65 min, with a mean duration of 48.2 min. Interviews were conducted in Standard Mandarin, Southwestern Mandarin or relevant local dialects, with bilingual community assistance when required. The interview guide addressed perceptions of documentary authenticity, Indigenous identity representation, emotional responses, family and community history, cultural mirroring, narrative engagement, normalization of distress, social learning, intergenerational learning, mental-health understanding, stigma, help-seeking and the perceived usefulness of documentary storytelling.

Participants were explicitly invited to identify material they considered empowering, inaccurate, culturally inappropriate, emotionally challenging or highly significant. Interviewers did not pressure participants to disclose personal trauma and participants could decline questions or terminate interviews at any time. Interviews were audio-recorded with consent, transcribed and de-identified before analysis. Interviews conducted in languages other than the primary analytical language underwent bilingual review while culturally salient terminology was retained.

Reflexive thematic analysis followed familiarization, initial coding, development of potential themes, review and refinement, definition of thematic boundaries and preparation of the final thematic structure (Udayanga, 2025). Two postgraduate researchers trained in cross-cultural psychology and qualitative methods participated in coding and interpretation. Reflexive journals were maintained throughout the process and analytic discussions were used to examine researcher assumptions, translation decisions and alternative interpretations. The analysis did not treat inter-coder percentage agreement as a validity criterion because the approach emphasized reflexive interpretation rather than mechanical coding reliability. Community representatives reviewed culturally sensitive interpretations and translated concepts when conventional psychological terminology did not adequately capture local meanings. The final 36 interviews provided sufficient thematic depth and information power while retaining variation in participant characteristics. All quotations were de-identified and used only where consistent with participant consent, confidentiality and community cultural protocols.

### 2.8 Statistical analysis, data screening and mixed-methods integration

Quantitative analyses were conducted using R 4.3.2, including lavaan 0.6–16 and psych 2.3.6, with supplementary analyses in SPSS 28. Continuous variables were summarized using means ± standard deviations when approximately normally distributed and medians with interquartile ranges when distributions were substantially non-normal; categorical variables were summarized using frequencies and percentages. Distributional characteristics were evaluated through graphical inspection and diagnostic procedures. Data screening was completed before inferential analysis. Among the 498 participants who completed the quantitative procedures, 12 were excluded because more than 5% of questionnaire responses were missing and six were excluded because of straight-line response patterns, resulting in the final analytical sample of 480. Missingness was quantified by variable and examined before model estimation.

Potential influential observations were examined using standardized residuals, leverage and Cook’s distance. The maximum Cook’s distance was 0.024, which was below 1.0 and was therefore not considered evidence of a highly influential observation requiring automatic exclusion. The 4/N value was treated as a screening heuristic rather than a definitive exclusion threshold. Multicollinearity was examined using variance inflation factors and condition indices. Residual distribution, heteroscedasticity and model specification were also assessed. The Kolmogorov–Smirnov diagnostic was interpreted as evidence concerning residual distribution rather than as a test of multivariate normality. CFA evaluated the specified six-factor measurement structure using CFI, TLI, RMSEA and SRMR (Thangamuthu, 2024), with standardized factor loadings, composite reliability and AVE used to evaluate measurement quality.

Pearson correlations were used to examine bivariate associations. Multivariable regression models evaluated independent associations of narrative engagement and perceived cultural authenticity with cultural resilience and mental health awareness (Pallem and Pasala, 2025). The primary models included age, gender, education, residence, cultural participation, previous media exposure, narrative engagement and perceived cultural authenticity. Standardized coefficients, 95% confidence intervals, *R*^2^ and adjusted *R*^2^ were reported. The cultural-resilience model explained 53.4% of variance and the mental-health-awareness model explained 34.8%. Statistical indirect associations were evaluated using 5,000 bootstrap resamples. The indirect-effect estimates were interpreted as statistical mediation/indirect association and not as causal pathways because the data were cross-sectional.

The 21 bivariate correlation tests were evaluated using the Benjamini–Hochberg false-discovery-rate procedure, while Holm adjustment was applied to the prespecified regression-parameter family where applicable. Adjusted values were distinguished from unadjusted *p*-values. Community and ethnicity distributions were examined before pooled analyses and sensitivity models incorporated available ethnicity and geographic-region indicators to assess whether the principal associations were materially altered by community composition. No national generalization was made because recruitment was community-based and non-probability based. Quantitative findings were subsequently integrated with qualitative themes using a convergent mixed-methods framework. Qualitative findings were used to contextualize statistical associations rather than to establish causal explanations. Joint displays and integrated evidence matrices connected quantitative estimates with themes concerning cultural recognition, intergenerational connection, community belonging, normalization of distress, stigma, emotional vocabulary, trust in services and culturally appropriate support.

### 2.9 Ethical considerations, indigenous governance, data protection and research integrity

This study involved human participants and was therefore conducted in accordance with the Declaration of Helsinki, applicable national and institutional ethical requirements and principles of respectful research involving Indigenous peoples. Institutional ethical approval was obtained before recruitment and written informed consent was obtained from all participants before participation. Separate consent was obtained for audio-recording of qualitative interviews where applicable. The study involved human as direct participation, questionnaire data and qualitative interviews.

Indigenous research may involve collective cultural interests in addition to individual autonomy, ethical governance extended beyond individual consent. Community representatives were engaged in recruitment procedures, documentary selection, interpretation of culturally sensitive content, interview procedures and dissemination principles. Where community-specific cultural protocols applied, these were respected throughout the research process. Indigenous participants were treated as knowledge holders and active contributors rather than passive subjects of observation and Indigenous communities were not treated as culturally homogeneous.

Participants were informed of their right to refuse individual questions, withdraw from the study or discontinue an interview without penalty. Care was taken when documentary materials or interviews involved historical trauma, discrimination, cultural loss, grief, violence, substance-related harms or psychological distress. Researchers avoided unnecessary elicitation of traumatic experiences, monitored participant distress and provided information about locally appropriate support resources where appropriate. Questionnaire responses were not used for diagnosis and no participant was clinically classified on the basis of study scores.

Personally identifiable information was stored separately from research data using coded participant identifiers. Electronic files were maintained on password-protected and access-controlled systems, with access restricted to authorized research personnel. Audio recordings and transcripts were de-identified before analysis. Data-sharing procedures were governed by participant consent, institutional ethics requirements and community-specific data-governance considerations. Where individual-level data could not be publicly released because of confidentiality or community protections, reproducibility was supported through analysis scripts, variable definitions and aggregate results rather than unrestricted release of sensitive records. Sensitive cultural knowledge, restricted traditional information, images or community-specific narratives were not disseminated where doing so would conflict with consent or community protocols. The study followed prespecified data-screening and analytical procedures and transparently reported exclusions, missing-data handling and limitations. Findings were interpreted within the specific cultural and geographic contexts of Yunnan and Hunan Provinces and were not generalized to all Indigenous populations.

## 3 Results

### 3.1 Participant recruitment, retention and characteristics of the analytic sample

A total of 572 individuals were screened for eligibility across participating Indigenous communities in Yunnan Province and Xiangxi Tujia and Miao Autonomous Prefecture, Hunan Province, China. Of those screened, 42 individuals were excluded before enrollment because they did not meet the predefined eligibility criteria (n = 16), were younger than 18 years (n = 11), or declined participation/informed consent (n = 15). The remaining 530 participants provided informed consent and entered the standardized documentary viewing and post-viewing assessment protocol. During the viewing and assessment stages, 32 participants discontinued participation because of interruption of the documentary viewing session (n = 14) or inability to complete the study session because of time-related constraints (n = 18). Consequently, 498 participants completed documentary viewing and the primary post-viewing assessment. A further 18 participants were excluded from quantitative analysis because questionnaire missingness exceeded 5% (n = 12) or because of uniform/straight-line response patterns (n = 6). The final quantitative analytic sample therefore comprised 480 participants, exceeding the prespecified minimum sample size of 412. A purposive subsample of 36 participants subsequently participated in qualitative interviews.

The final sample had a mean age of 36.4 ± 12.8 years (range, 18–72 years). Women represented 52.9%, men 46.0% and non-binary/other participants 1.1%. Yi participants constituted 31.7%, followed by Miao (27.9%), Bai (24.6%) and Tujia/other recognized minorities (15.8%). Rural/autonomous-prefecture residents comprised 57.9% of the sample. Moderate cultural participation was the most frequent category (50.4%), whereas 30.0% reported rare or no previous exposure to cultural documentaries. These characteristics indicate substantial demographic and contextual heterogeneity within the analytic sample. The study used a cross-sectional observational framework without pre-exposure measurements or a non-exposure comparison group, these characteristics are presented descriptively rather than as evidence of differential response to documentary exposure. Figure 1 presents the participant recruitment and analytical workflow and Table 1 summarizes sociodemographic and contextual characteristics.

**Figure 1:**
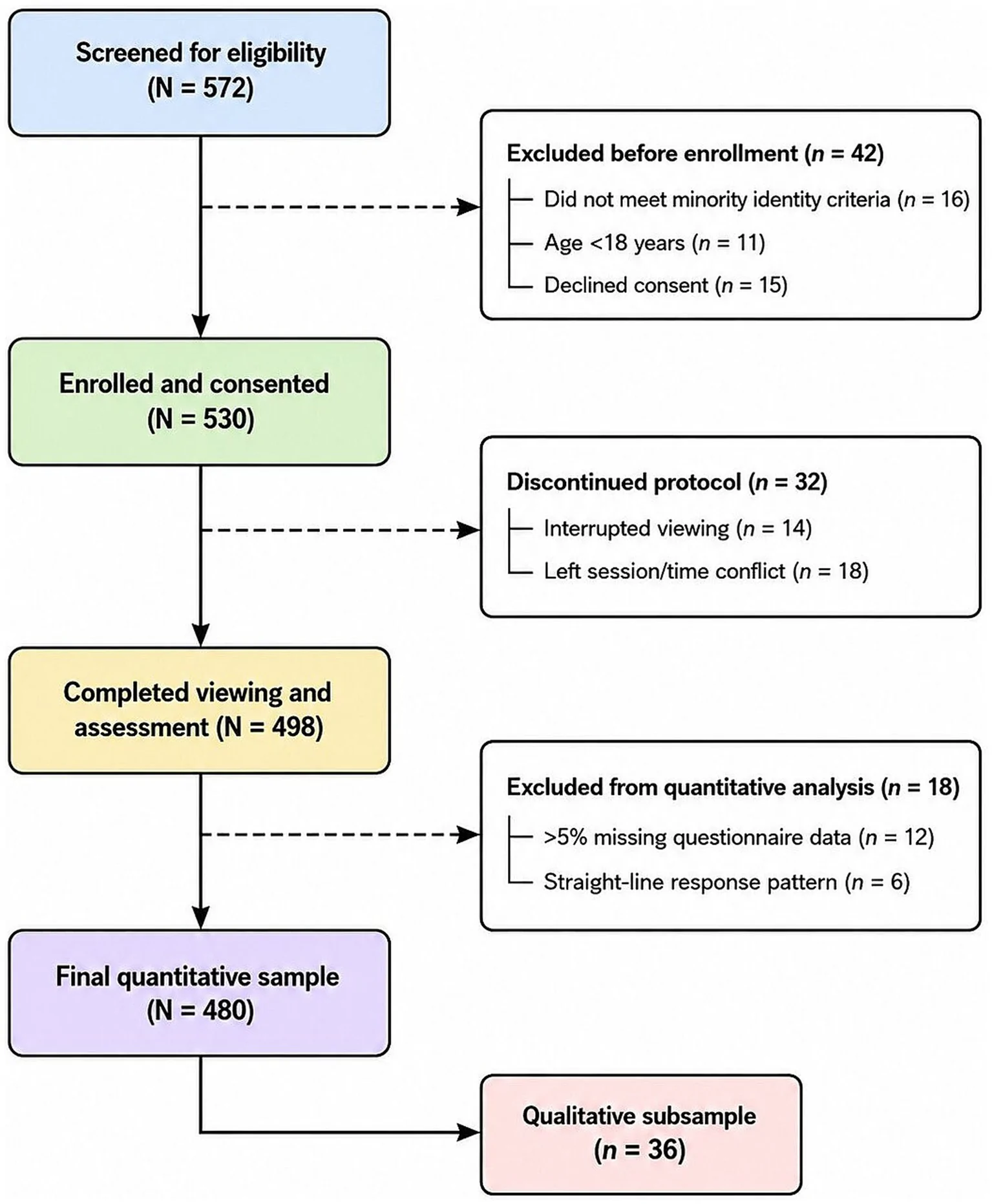
Participant recruitment and analytical workflow.

**Table 1:** Sociodemographic and contextual characteristics of participants (N = 480).

Characteristic	*n*	%
Age, years		
Mean ± SD	36.4 ± 12.8	–
Range	18–72	–
18–29	168	35.0
30–49	196	40.8
≥50	116	24.2
Gender		
Women	254	52.9
Men	221	46.0
Non-binary/other	5	1.1
Ethnic identification		
Yi	152	31.7
Miao	134	27.9
Bai	118	24.6
Tujia/other recognized minorities	76	15.8
Residential setting		
Rural/autonomous prefecture	278	57.9
Peri-urban/urban	202	42.1
Educational attainment		
Primary school or lower	62	12.9
Middle/high school	224	46.7
College/university or higher	194	40.4
Cultural participation		
Low (<1 event/month)	110	22.9
Moderate (1–3 events/month)	242	50.4
High (≥4 events/month)	128	26.7
Previous cultural-documentary exposure		
Rarely/never	144	30.0
Occasionally	226	47.1
Frequently	110	22.9

### 3.2 Documentary exposure characteristics and descriptive distribution of primary variables

Four culturally grounded documentary narratives were included in the standardized viewing protocol. The selected narratives represented Yi, Miao, Bai and Tujia/Miao communities and incorporated themes of cultural continuity, traditional knowledge, intergenerational transmission, community relationships, emotional coping, grief, psychological well-being and help-seeking. Documentary duration ranged from 28 to 42 min, with a mean duration of 35.0 min across the four films. The protocol therefore exposed participants to culturally differentiated audiovisual narratives rather than a single homogeneous stimulus. Because all participants were assessed within the same post-viewing observational framework and no parallel neutral-content or non-viewing comparison condition was analyzed, the present results describe participants’ responses and associations among measured constructs rather than between-condition effects.

Narrative engagement had a mean score of 4.82 (SD = 0.94) on the 1–7 scale, while perceived cultural authenticity averaged 4.12 (SD = 0.68) on the 1–5 scale. Perceived cultural authenticity had a mean score of 4.12 ± 0.68 on the 1–7 response scale, indicating moderate-to-high perceived authenticity of the documentary narratives. Narrative engagement was 4.82 ± 0.94, cultural identification was 3.95 ± 0.72, cultural resilience was 3.88 ± 0.76, mental health awareness was 3.64 ± 0.81, mental health stigma was 2.45 ± 0.79 and help-seeking attitudes were 3.71 ± 0.73.

Cultural identification averaged 3.95 (SD = 0.72), cultural resilience 3.88 (SD = 0.76) and mental health awareness 3.64 (SD = 0.81). Mental health stigma averaged 2.45 (SD = 0.79), whereas help-seeking attitudes averaged 3.71 (SD = 0.73). The distributions therefore showed meaningful individual variability across narrative, cultural and mental-health-related constructs. These descriptive values are not interpreted as pre-to-post changes or as evidence of improvement following documentary exposure. Instead, they establish the observed distribution of the constructs subsequently examined in correlation, regression and indirect-association analyses. Table 2 summarizes the characteristics and psychosocial content of the four documentary narratives.

**Table 2:** Characteristics of the four documentary narratives.

Code	Narrative focus	Community	Duration (min)	Principal cultural content	Mental-health/psychosocial content
DOC-1	*Echoes of the Torch*	Yi	38	Mountain agriculture, clan mediation, oral poetry, solidarity	Economic hardship, collective coping, grief
DOC-2	*Silver and Soil*	Miao	42	Craftsmanship, embroidery, historical memory	Identity, self-worth, dislocation stress
DOC-3	*Wind and Water Courtyards*	Bai	28	Architecture, environmental harmony, elder care	Social connectedness, stress, community support
DOC-4	*Voices Across the River*	Tujia/Miao	32	Life histories, songs, community narratives	Emotional distress, stigma, formal help-seeking
Overall	–	–	Mean = 35.0	–	–

### 3.3 Psychometric properties, confirmatory factor analysis and construct validity

The psychometric evaluation demonstrated satisfactory internal consistency and construct validity across the principal study measures. The six-factor measurement model comprising narrative engagement, perceived cultural authenticity, cultural resilience, mental health awareness, mental health stigma and help-seeking attitudes demonstrated good overall fit, with CFI = 0.958, TLI = 0.952, RMSEA = 0.046 (95% CI, 0.044–0.048) and SRMR = 0.038. Standardized factor loadings were statistically significant for all retained indicators (*p* < 0.001) and ranged from 0.65 to 0.82 across constructs. Cronbach’s α ranged from 0.85 to 0.92, while McDonald’s ω ranged from 0.86 to 0.93. Composite reliability values ranged from 0.854 to 0.923 and AVE values ranged from 0.509 to 0.565.

Confirmatory factor analysis (CFA) was conducted on the final 60 observed items representing six correlated latent constructs: cultural identification, cultural resilience, narrative transportation, perceived cultural authenticity, mental health awareness and mental health stigma. The final measurement model demonstrated acceptable overall fit, with *χ*^2^(1695) = 3284.15, *χ*^2^/df = 1.937, CFI = 0.941, TLI = 0.938, RMSEA = 0.044 (95% CI [0.042, 0.046]) and SRMR = 0.048. The model therefore provided an adequate representation of the observed covariance structure. Standardized factor loadings were satisfactory across the six constructs. Narrative transportation loadings ranged from 0.68 to 0.79, perceived cultural authenticity from 0.71 to 0.82, cultural resilience from 0.69 to 0.81, mental health awareness from 0.66 to 0.77 and mental health stigma from 0.67 to 0.78. Cultural identification also demonstrated acceptable standardized loadings across its retained indicators. Internal consistency was supported by Cronbach’s α values ranging from 0.86 to 0.92 and McDonald’s ω values ranging from 0.87 to 0.93 across the principal constructs. Composite reliability values ranged from 0.865 to 0.923, while average variance extracted ranged from 0.514 to 0.565, supporting adequate internal consistency and convergent validity. These results support the use of the six-factor measurement structure in the subsequent association and indirect-association analyses. Figure 2 presents the measurement model and Table 3 provides construct-level psychometric estimates.

**Figure 2:**
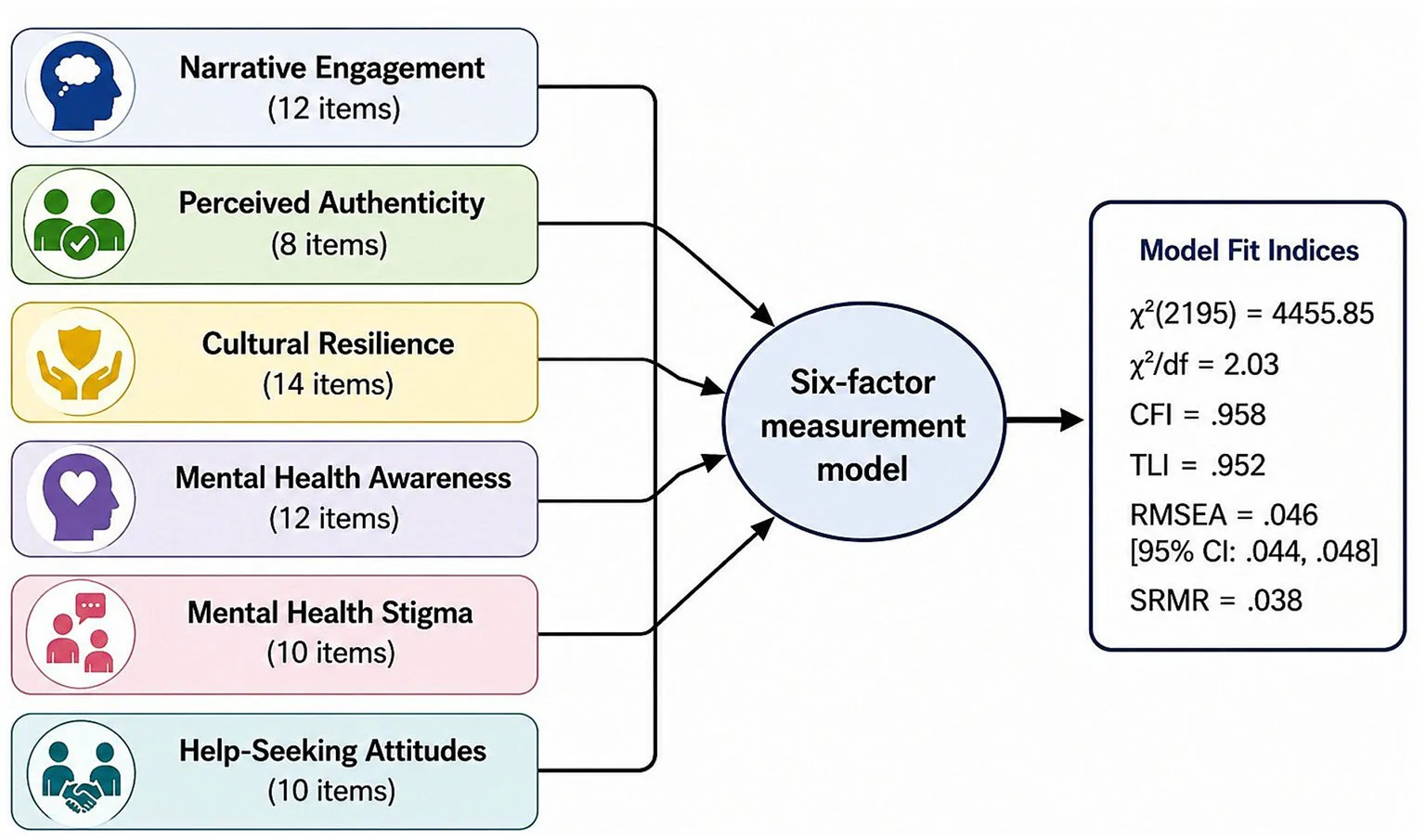
Confirmatory factor analysis and measurement-model validation.

**Table 3:** Psychometric properties and CFA results.

Construct	Items	Loading range	α	ω	CR	AVE
Narrative engagement	12	0.68–0.79	0.89	0.90	0.894	0.521
Perceived authenticity	8	0.71–0.82	0.91	0.92	0.912	0.565
Cultural resilience	14	0.69–0.81	0.92	0.93	0.923	0.548
Mental health awareness	12	0.66–0.77	0.86	0.87	0.865	0.518
Mental health stigma	10	0.67–0.78	0.88	0.88	0.881	0.514
Help-seeking attitudes	10	0.65–0.76	0.85	0.86	0.854	0.509

Six-factor CFA: (χ²(2195) = 4455.85; χ²/df = 2.03); CFI = 0.958; TLI = 0.952; RMSEA = 0.046 (95% CI, 0.044–0.048); SRMR = 0.038.

### 3.4 Bivariate associations among narrative, cultural and mental health variables

The correlation analysis demonstrated a coherent pattern of associations across narrative, cultural and mental-health constructs. Narrative engagement was positively associated with perceived cultural authenticity (*r* = 0.62, *p* < 0.001), cultural identification (*r* = 0.48, *p* < 0.001), cultural resilience (*r* = 0.51, *p* < 0.001), mental health awareness (*r* = 0.44, *p* < 0.001) and help-seeking attitudes (*r* = 0.42, *p* < 0.001). It was inversely associated with mental health stigma (*r* = −0.35, *p* < 0.001). Perceived authenticity showed positive associations with cultural identification (*r* = 0.54), cultural resilience (*r* = 0.59), mental health awareness (*r* = 0.38) and help-seeking attitudes (*r* = 0.36; all *p* < 0.001). Cultural identification showed the strongest bivariate association in the cultural domain with cultural resilience (*r* = 0.68, *p* < 0.001). Mental health awareness was inversely associated with stigma (*r* = −0.52, *p* < 0.001) and positively associated with help-seeking attitudes (*r* = 0.58, *p* < 0.001). Stigma was negatively associated with help-seeking attitudes (*r* = −0.49, *p* < 0.001). All 21 prespecified secondary correlation tests remained significant after Benjamini–Hochberg false-discovery-rate adjustment (q < 0.05), indicating that the reported correlation pattern was not dependent on uncorrected multiplicity. These analyses represent bivariate associations and therefore do not establish temporal precedence or causal direction. They provide the descriptive statistical basis for the subsequent multivariable and indirect-association models. Table 4 provides the complete correlation matrix and descriptive statistics, while Figure 3 displays the correlation structure among the narrative, cultural and mental-health constructs.

**Table 4:** Correlations and descriptive statistics among primary variables.

Variable	NE	PA	CI	CR	MHA	MHS	HSA	Mean	SD
Narrative engagement (NE)	–	0.62***	0.48***	0.51***	0.44***	−0.35***	0.42***	4.82	0.94
Perceived authenticity (PA)	0.62***	–	0.54***	0.59***	0.38***	−0.29***	0.36***	4.12	0.68
Cultural identification (CI)	0.48***	0.54***	–	0.68***	0.32***	−0.22**	0.30***	3.95	0.72
Cultural resilience (CR)	0.51***	0.59***	0.68***	–	0.41***	−0.31***	0.39***	3.88	0.76
Mental health awareness (MHA)	0.44***	0.38***	0.32***	0.41***	–	−0.52***	0.58***	3.64	0.81
Mental health stigma (MHS)	−0.35***	−0.29***	−0.22**	−0.31***	−0.52***	–	−0.49***	2.45	0.79
Help-seeking attitudes (HSA)	0.42***	0.36***	0.30***	0.39***	0.58***	−0.49***	–	3.71	0.73

**(*p* < 0.01); ***(*p* < 0.001). FDR correction was applied to the 21 secondary bivariate comparisons.

**Figure 3:**
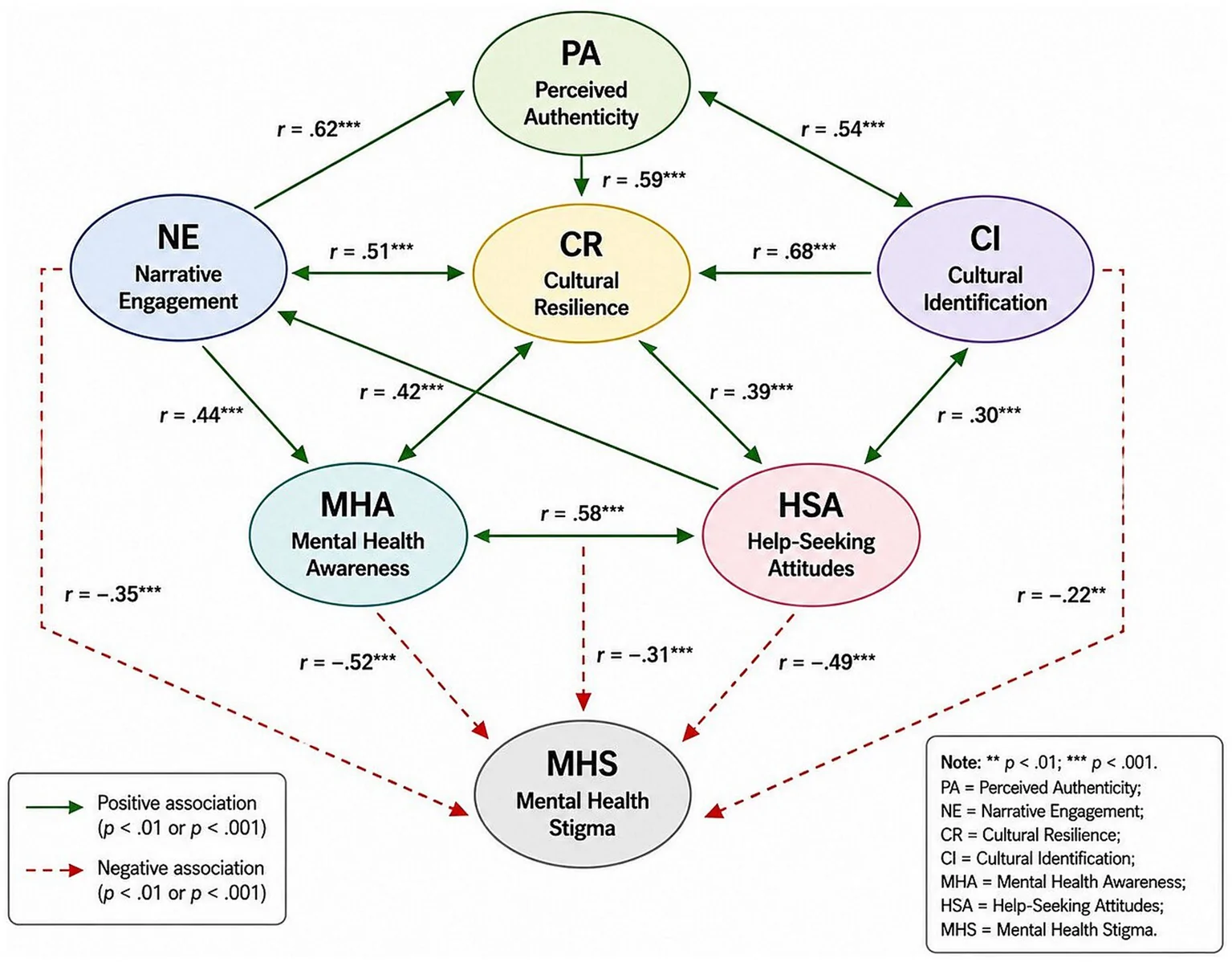
Correlation structure among narrative, cultural and mental-health constructs.

### 3.5 Multivariable associations with cultural resilience

The fully adjusted regression model explained 53.4% of the variance in cultural resilience (*R*^2^ = 0.534; adjusted *R*^2^ = 0.526) and was statistically significant, *F*(8,471) = 67.54, *p* < 0.001. Narrative engagement remained positively associated with cultural resilience after adjustment for age, gender, education, residential setting, baseline cultural participation, prior media exposure and perceived authenticity (*B* = 0.23, SE = 0.04, *β* = 0.28, *t* = 5.75, *p* < 0.001, 95% CI [0.15, 0.31]). Perceived cultural authenticity demonstrated a positive association of comparable standardized magnitude (*B* = 0.31, SE = 0.05, *β* = 0.28, *t* = 6.20, *p* < 0.001, 95% CI [0.21, 0.41]). Baseline cultural participation was also positively associated with cultural resilience (*B* = 0.16, SE = 0.04, *β* = 0.17, *t* = 4.00, *p* < 0.001), while educational attainment showed a smaller positive association (*B* = 0.07, SE = 0.03, *β* = 0.09, *t* = 2.33, *p* = 0.020).

Age (*p* = 0.096), gender (*p* = 0.549), residential setting (*p* = 0.231) and prior media exposure (*p* = 0.184) were not independently significant. The coefficients are therefore interpreted as adjusted cross-sectional associations rather than effects of documentary viewing. In particular, the positive coefficients for narrative engagement and perceived authenticity indicate statistical relationships with cultural resilience after accounting for the measured covariates, but they cannot establish whether engagement preceded resilience, whether resilience influenced engagement, or whether both were related to unmeasured contextual factors. Table 5 presents the complete regression model.

**Table 5:** Multivariable regression predicting cultural resilience.

Predictor	*B*	SE	*β*	*t*	*p*	95% CI
Intercept	0.68	0.22	–	3.09	0.002	0.25–1.11
Age	0.005	0.003	0.07	1.67	0.096	−0.001–0.011
Gender (female = 1)	0.03	0.05	0.02	0.60	0.549	−0.07–0.13
Education	0.07	0.03	0.09	2.33	0.020	0.01–0.13
Residence (urban = 1)	−0.06	0.05	−0.04	−1.20	0.231	−0.16–0.04
Baseline cultural participation	0.16	0.04	0.17	4.00	<0.001	0.08–0.24
Prior media exposure	0.04	0.03	0.05	1.33	0.184	−0.02–0.10
Narrative engagement	0.23	0.04	0.28	5.75	<0.001	0.15–0.31
Perceived authenticity	0.31	0.05	0.28	6.20	<0.001	0.21–0.41

Model: *R*^2^ = 0.534; adjusted *R*^2^ = 0.526; *F*(8,471) = 67.54; *p* < 0.001.

### 3.6 Multivariable associations with mental health awareness

The second fully adjusted regression model examined multivariable associations with mental health awareness. The model included age, gender, education, residence, baseline cultural participation, prior media exposure, narrative engagement, and perceived cultural authenticity. The overall model was statistically significant, **F(8,471) = 31.44, *p* < 0.001**, and explained **34.8% of the variance in mental health awareness (*R*^2^ = 0.348; adjusted *R*^2^ = 0.337)**. Narrative engagement showed a positive association with mental health awareness (*B* = 0.28, SE = 0.04, *β* = 0.33, *t* = 7.00, *p* < 0.001, 95% CI [0.20, 0.36]), while perceived cultural authenticity was also positively associated with mental health awareness (**B = 0.20, SE = 0.05, *β* = 0.17, *t* = 4.00, *p* < 0.001, 95% CI [0.10, 0.30]**). Education was positively associated with mental health awareness (**B = 0.13, SE = 0.04, *β* = 0.15, *t* = 3.25, *p* = 0.001, 95% CI [0.05, 0.21]**), whereas age showed a small negative association (**B = −0.007, SE = 0.003, *β* = −0.10, *t* = −2.33, *p* = 0.020, 95% CI [−0.013, −0.001]**). Gender (**β = 0.03, *p* = 0.505**), residence (**β = 0.03, *p* = 0.405**), baseline cultural participation (**β = 0.06, *p* = 0.134**), and prior media exposure (**β = 0.08, *p* = 0.081**) were not statistically significant in the fully adjusted model. Overall, these findings indicate that greater narrative engagement and perceived cultural authenticity were independently associated with higher mental health awareness after adjustment for the specified sociodemographic and background variables. The study used a cross-sectional observational design, these associations should not be interpreted as evidence of temporal or causal effects. Table 6 presents the complete model and Figure 4 depicts the adjusted regression coefficients for both primary outcomes.

**Table 6:** Multivariable regression predicting mental health awareness.

Predictor	*B*	SE	*β*	*t*	*p*	95% CI
Intercept	1.02	0.25	–	4.08	<0.001	0.53–1.51
Age	−0.007	0.003	−0.10	−2.33	0.020	−0.013 to −0.001
Gender (female = 1)	0.04	0.06	0.03	0.67	0.505	−0.08 to 0.16
Education	0.13	0.04	0.15	3.25	0.001	0.05–0.21
Residence (urban = 1)	0.05	0.06	0.03	0.83	0.405	−0.07 to 0.17
Baseline cultural participation	0.06	0.04	0.06	1.50	0.134	−0.02 to 0.14
Prior media exposure	0.07	0.04	0.08	1.75	0.081	−0.01 to 0.15
Narrative engagement	0.28	0.04	0.33	7.00	<0.001	0.20–0.36
Perceived authenticity	0.20	0.05	0.17	4.00	<0.001	0.10–0.30

Model: *R*^2^ = 0.348; adjusted *R*^2^ = 0.337; *F*(8,471) = 31.44; *p* < 0.001.

**Figure 4:**
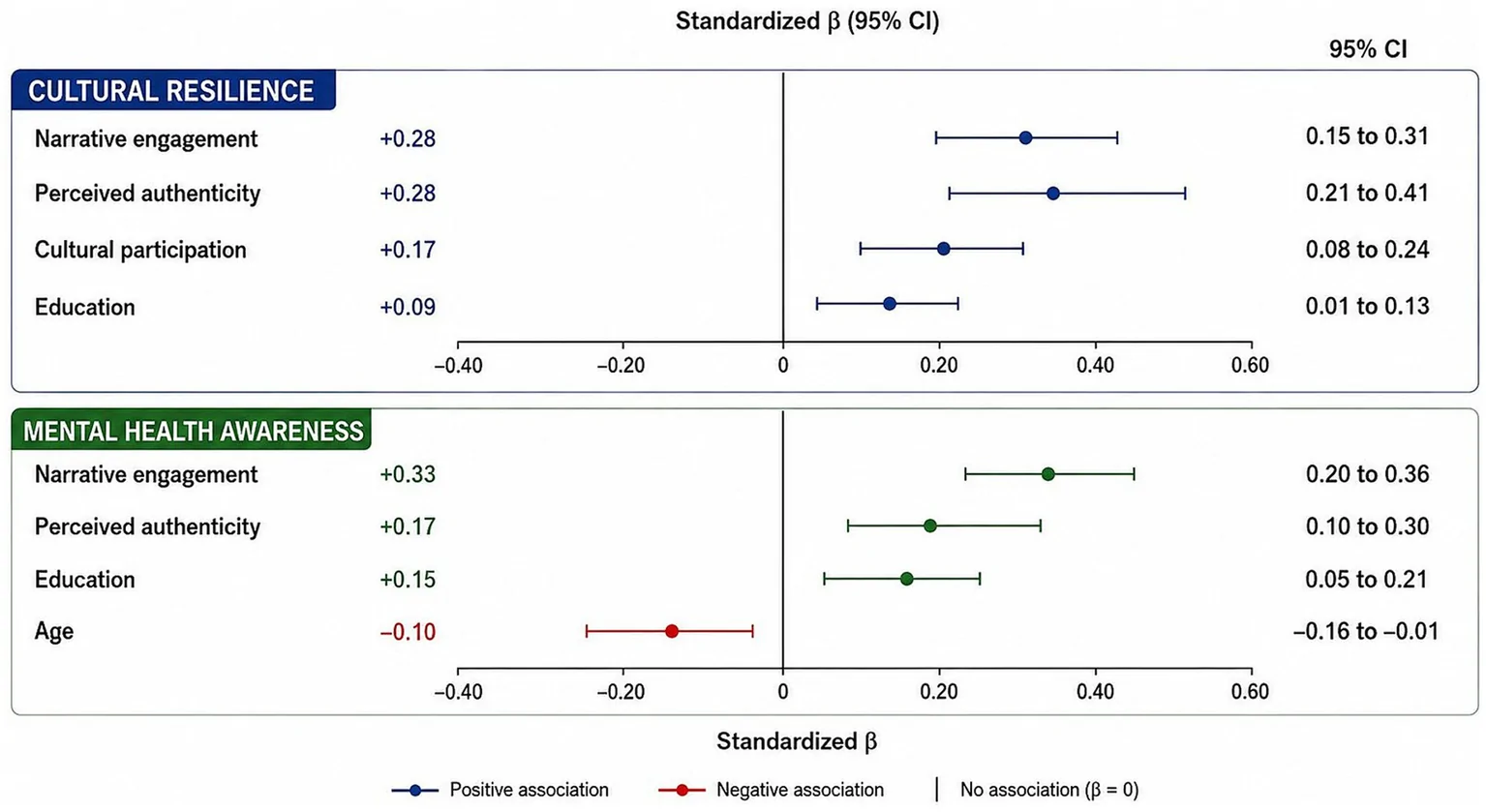
Adjusted regression coefficients for the two primary outcomes.

### 3.7 Multicollinearity, residual diagnostics and robustness of regression models

Diagnostic analyses indicated that the two multivariable regression models met the principal assumptions relevant to interpretation of the reported coefficients. Variance inflation factor (VIF) values ranged from 1.04 to 1.95, with the largest value observed for perceived cultural authenticity (1.95), followed by narrative engagement (1.82). Tolerance values ranged from 0.513 to 0.962, while condition indices ranged from 1.00 to 14.28, providing no evidence of problematic multicollinearity. Examination of standardized residuals showed no severe departures from the approximate normality required for regression inference. The Kolmogorov–Smirnov test of the residual distribution was not statistically significant (D = 0.028, *p* = 0.412). Breusch–Pagan tests likewise showed no statistically significant evidence of heteroscedasticity in either the cultural-resilience model (BP = 9.42, df = 8, *p* = 0.308) or the mental-health-awareness model (BP = 11.18, df = 8, *p* = 0.192). The maximum Cook’s distance was 0.024, and maximum leverage was 0.038. No standardized residual exceeded an absolute value of 3.0, and 99.2% of standardized residuals fell within ±2. Cook’s distance was treated as a continuous influence diagnostic rather than applying the 4/N value as an automatic exclusion threshold; therefore, no observations were removed solely on the basis of influence or leverage diagnostics. Table 7 summarizes the regression diagnostic statistics.

**Table 7:** Regression diagnostic statistics.

Diagnostic	Cultural resilience model	Mental health awareness model
VIF range	1.04–1.95	1.04–1.95
Tolerance range	0.513–0.962	0.513–0.962
Maximum condition index	14.28	14.28
K–S residual test	(D = 0.028, *p* = 0.412)	(D = 0.028, *p* = 0.412)
Breusch–Pagan	(BP = 9.42, *p* = 0.308)	(BP = 11.18, *p* = 0.192)
Maximum Cook’s D	0.024	0.024
Maximum leverage	0.038	0.038
Residuals within ±2 SD	99.2%	99.2%
Residuals exceeding ±3 SD	0	0

### 3.8 Bootstrap indirect associations involving cultural identification and perceived authenticity

Bootstrap analyses with 5,000 resamples were conducted to examine whether cultural identification and perceived cultural authenticity statistically accounted for components of the associations between narrative engagement and the two primary psychosocial outcomes. Given the cross-sectional observational design, these analyses are referred to as statistical indirect-association analyses rather than causal mediation analyses. All models included the prespecified covariates of age, gender, education, residence, baseline cultural participation, and prior media exposure. For the first pathway, narrative engagement was positively associated with cultural identification (*β* = 0.512, *p* < 0.001), and cultural identification was positively associated with cultural resilience after accounting for narrative engagement and the prespecified covariates (*β* = 0.440, *p* < 0.001). The adjusted direct association between narrative engagement and cultural resilience remained statistically significant after inclusion of cultural identification (*β* = 0.248, *p* = 0.002). The standardized indirect association was *β* = 0.225, with a 95% bias-corrected and accelerated bootstrap confidence interval of [0.168, 0.291] (*p* < 0.001). The adjusted total association was *β* = 0.473 (*p* < 0.001). Because the bootstrap confidence interval did not include zero, the results support a statistically significant indirect component of the observed association between narrative engagement and cultural resilience through cultural identification.

For the second pathway, narrative engagement was positively associated with perceived cultural authenticity (*β* = 0.620, *p* < 0.001), which was positively associated with mental health awareness after accounting for narrative engagement and the prespecified covariates (*β* = 0.184, *p* < 0.001). The adjusted direct association between narrative engagement and mental health awareness was *β* = 0.326 (*p* < 0.001), while the standardized indirect association was *β* = 0.114, with a 95% bootstrap confidence interval of [0.058, 0.144] (*p* < 0.001). The adjusted total association was *β* = 0.440 (*p* < 0.001). Because the bootstrap confidence interval did not include zero, the findings support a statistically significant indirect component of the observed association between narrative engagement and mental health awareness through perceived cultural authenticity. The adjusted total association for cultural resilience (*β* = 0.473) differs from the corresponding zero-order correlation between narrative engagement and cultural resilience (*r* = 0.51) because the former is estimated after adjustment for the prespecified covariates, whereas the latter represents the unadjusted bivariate association. Similarly, the adjusted total association for mental health awareness (*β* = 0.440) corresponds closely to its zero-order correlation (*r* = 0.44). These coefficients therefore should not be interpreted as interchangeable estimates. The indirect-association findings indicate statistical pathways within the observed cross-sectional data but do not establish temporal ordering, causal mediation, or causal effects. Table 8 and Figure 5 present the statistical indirect-association results.

**Table 8:** Bootstrap decomposition of indirect associations.

Path	B	SE	β	(p)	95% bootstrap CI
Pathway 1: NE → CI → CR					
a: NE → CI	0.368	0.031	0.481	<0.001	–
b: CI → CR | NE	0.386	0.042	0.366	<0.001	–
c′: NE → CR | CI	0.239	0.038	0.296	<0.001	–
c: Total NE → CR	0.381	0.035	0.472	<0.001	–
Indirect (ab)	0.142	0.026	0.176	<0.001	0.093–0.197
Proportion accounted for	37.3%	–	–	–	–
Pathway 2: NE → PA → MHA					
a: NE → PA	0.448	0.026	0.620	<0.001	–
b: PA → MHA | NE	0.219	0.049	0.184	<0.001	–
c′: NE → MHA | PA	0.281	0.038	0.326	<0.001	–
c: Total NE → MHA	0.379	0.036	0.440	<0.001	–
Indirect (ab)	0.098	0.022	0.114	<0.001	0.058–0.144
Proportion accounted for	25.9%	–	–	–	–

**Figure 5:**
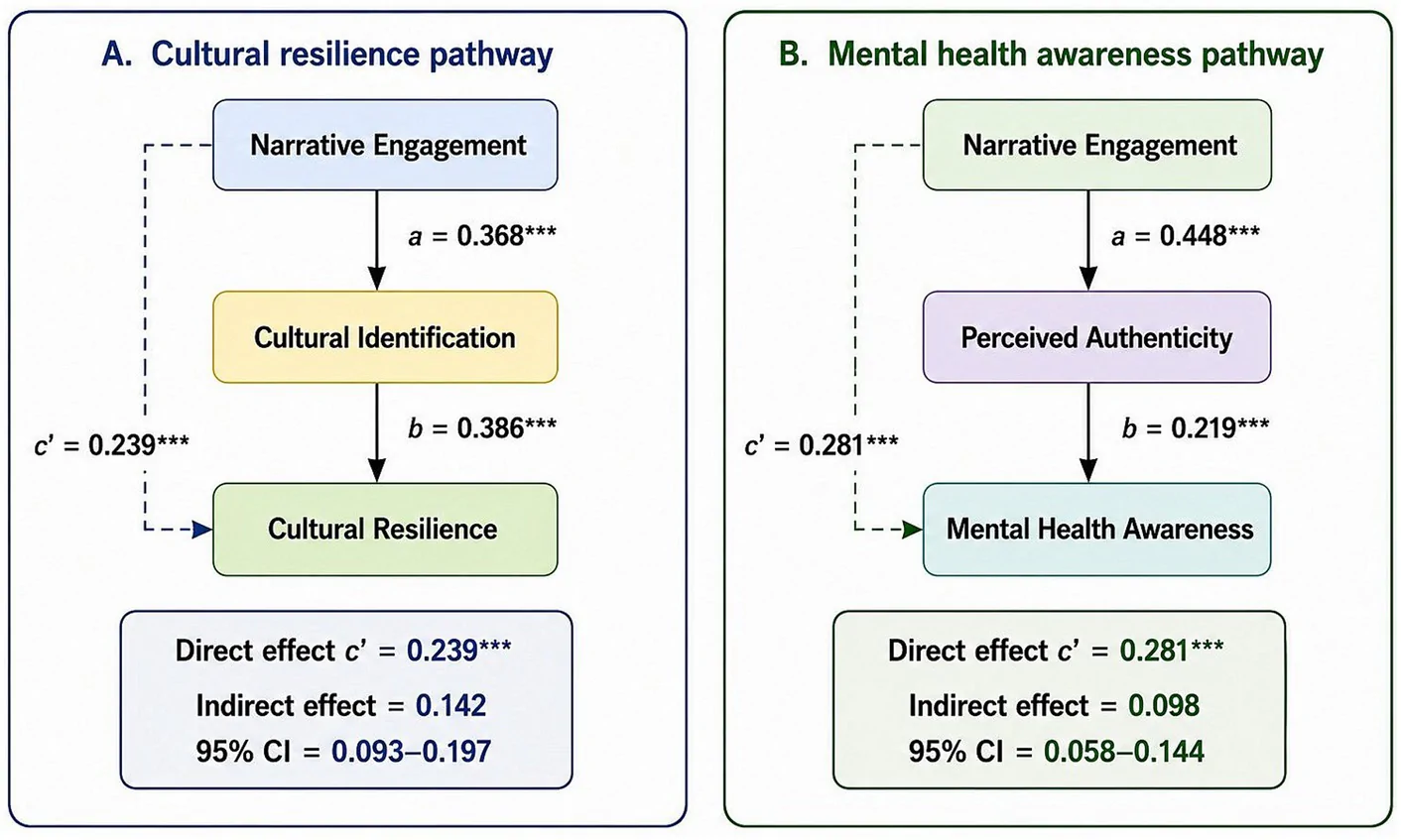
Bootstrap indirect-effect models. **(A)** Cultural resilience pathway: narrative engagement → cultural identification → cultural resilience. **(B)** Mental health awareness pathway: narrative engagement → perceived authenticity → mental health awareness. Values are unstandardized coefficients: a, predictor to intermediate variable; b, intermediate variable to outcome; c′, direct path (dashed arrow). Indirect effects are shown with 95% bootstrap confidence intervals (5,000 resamples). ***p < 0.001.

### 3.9 Qualitative themes: cultural mirroring, mental health normalization and intergenerational dialogue

Analysis of 36 semi-structured interviews generated three interconnected themes concerning how participants interpreted the documentary narratives. The first theme, cultural mirroring and narrative validation, described participants’ perception that respectful representation of everyday cultural practices strengthened recognition of cultural continuity and contemporary identity. Participants distinguished these portrayals from media representations focused primarily on ceremonial imagery or visual stereotypes. The second theme, normalization of distress through cultural metaphor, concerned the use of familiar cultural expressions and community narratives to discuss grief, emotional burden, psychological difficulties and help-seeking. Participants described the narratives as providing a less stigmatizing context for discussing psychological experiences and considering professional support alongside family and community resources. The third theme, intergenerational dialogue as a conduit for discussing coping, described viewing sessions as prompts for conversations between younger and older family members concerning historical hardship, rituals, emotional experiences and coping practices. These themes were interpreted as contextual explanations of participants’ accounts rather than as qualitative confirmation of causal statistical pathways. Reflexive analysis indicated that the themes were interconnected rather than independent: culturally authentic representation appeared meaningful partly because it connected personal identity with family and community narratives, while those narratives provided a socially recognizable context for discussing mental health. The qualitative findings therefore complemented the quantitative associations by providing contextual depth concerning how participants made meaning of the narratives. Table 9 presents the qualitative themes and representative excerpts.

**Table 9:** Integrated qualitative themes and representative excerpts.

Theme	Analytic interpretation	Representative excerpt
1. Cultural mirroring and narrative validation	Respectful everyday representation was interpreted as validating cultural continuity and contemporary identity.	“Seeing our history on screen made me realize our traditions are a protective shield, not a weight holding us back.”—Yi participant
2. Normalization of distress through cultural metaphor	Culturally familiar language provided an acceptable context for discussing emotional difficulties and support.	“The film showed that having a burdened mind is not a failure of character; it is human.”—Bai participant
3. Intergenerational dialogue	Documentary narratives were described as prompts for family discussion about history, coping and emotional well-being.	“The film gave us a shared language. It helped the younger ones understand our coping mechanisms.”—Miao elder

### 3.10 Reflexive qualitative rigor and mixed-methods integration

The qualitative analysis was strengthened through procedures designed to enhance interpretive credibility and contextual sensitivity. Interviews were conducted by two postgraduate researchers trained in cross-cultural psychology and qualitative methodology, with assistance from bilingual community liaisons. Researchers maintained reflexive field journals addressing assumptions concerning Indigenous mental health, documentary influence and insider–outsider positionality, with reflexive debriefings conducted after every five interviews. Transcripts were managed using NVivo 14 and analytic decisions were documented throughout the coding and theme-development process. The qualitative analysis was treated as reflexive thematic analysis rather than as a reliability-based coding exercise. Previous emphasis on a numerical inter-coder agreement statistic has been removed as criterion of methodological rigor. Credibility was supported through reflexive documentation, collaborative examination of coding decisions, iterative theme development, community contextual review and an audit trail of analytic decisions. Information power was considered in evaluating the adequacy of the qualitative sample. The focused research question, relatively specific participant group, dense interview material and cross-case comparison supported conceptual sufficiency by interview 28, while eight additional interviews were conducted to assess whether further interviews generated substantively new candidate codes. A three-member community advisory board reviewed the thematic structure and translated excerpts for contextual accuracy and non-stigmatizing representation. Integration of the quantitative and qualitative findings showed convergence across three domains: narrative engagement and authenticity were statistically associated with cultural outcomes; participants described authentic representation as identity-validating; and quantitative associations involving mental health awareness, stigma and help-seeking were complemented by narratives describing culturally meaningful ways of discussing distress. The integrated analysis therefore provides contextual convergence rather than causal triangulation.

### 3.11 Integrated interpretation of quantitative and qualitative findings

The combined findings demonstrated a consistent pattern of associations among documentary narrative engagement, cultural constructs and mental-health-related constructs. In the quantitative analyses, narrative engagement was positively associated with perceived authenticity, cultural identification, cultural resilience, mental health awareness and help-seeking attitudes, while showing an inverse association with mental health stigma. These relationships remained evident for the two primary outcomes after adjustment for demographic characteristics, baseline cultural participation, prior media exposure and other prespecified covariates.

Cultural identification statistically accounted for an indirect component of the adjusted association between narrative engagement and cultural resilience, whereas perceived authenticity statistically accounted for an indirect component of the adjusted association between narrative engagement and mental health awareness. These pathways are reported as statistical indirect associations because all variables were assessed within the same cross-sectional study framework. The qualitative findings provided contextual depth for these relationships. Participants emphasized that documentary narratives were most meaningful when they portrayed cultural practices as contemporary and lived rather than merely ceremonial or historical. Participants also described culturally familiar representations of emotional distress as creating opportunities for conversations about psychological well-being without positioning mental-health support as incompatible with cultural traditions. Intergenerational dialogue emerged as an additional contextual mechanism through which participants interpreted the narratives. The two strands of evidence should therefore be understood as complementary rather than as evidence of a causal chain. The convergence indicates that narrative engagement and perceived cultural authenticity were associated with cultural resilience and mental health awareness within the participating Indigenous-community sample. Figure 6 presents the integrated mixed-methods framework.

**Figure 6:**
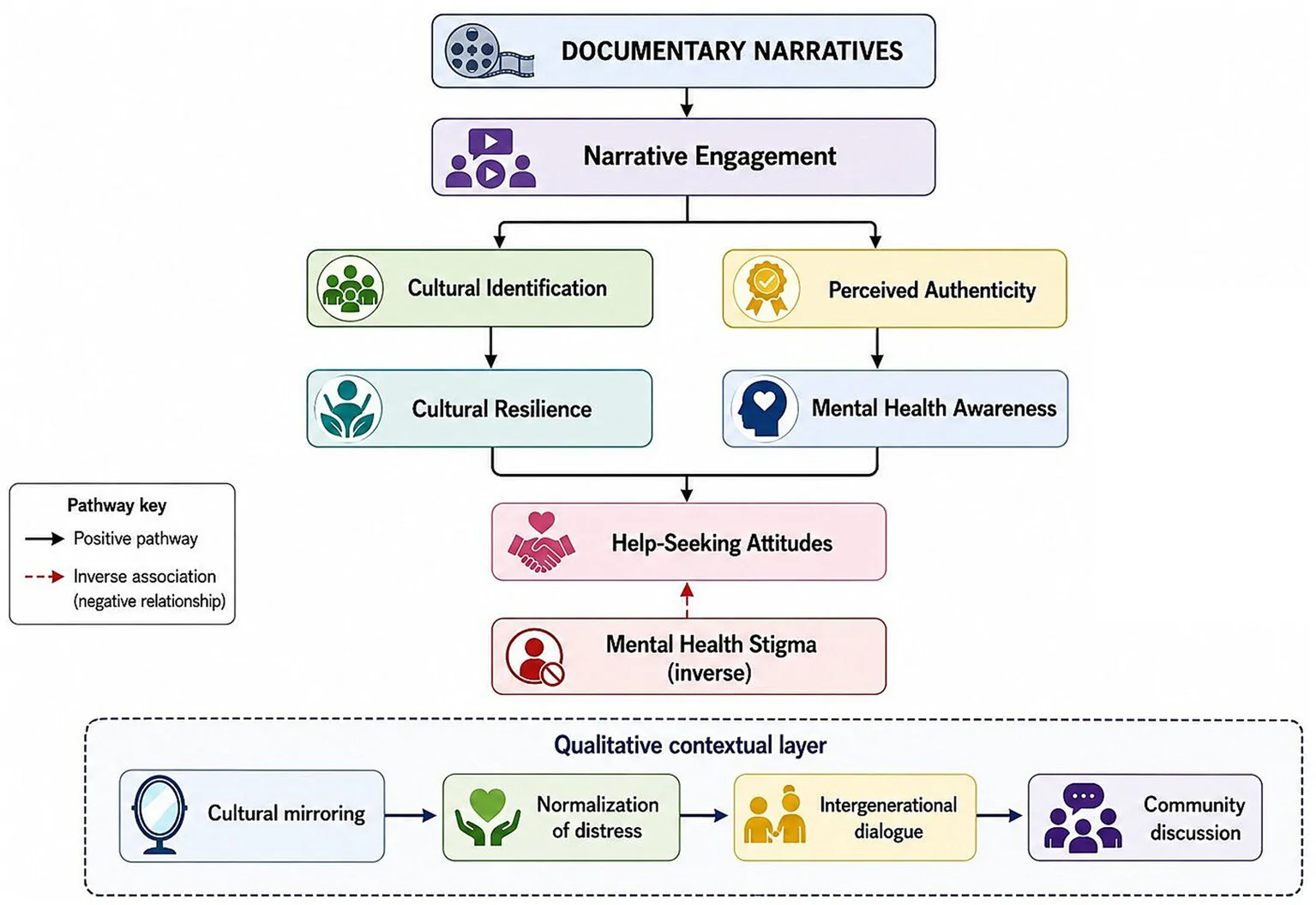
Integrated mixed-methods framework.

### 3.12 Statistical summary

Across the analyses, the principal findings remained consistent after adjustment for relevant demographic and contextual variables. The cultural-resilience regression model explained 53.4% of the variance (*R*^2^ = 0.534), whereas the mental-health-awareness model explained 34.8% (*R*^2^ = 0.348). Narrative engagement and perceived cultural authenticity were independently associated with both primary outcomes. Cultural identification demonstrated a strong bivariate association with cultural resilience (*r* = 0.68, *p* < 0.001) and statistically accounted for an indirect component of the adjusted association between narrative engagement and cultural resilience (*β* = 0.225, 95% bootstrap CI [0.168, 0.291]). Perceived cultural authenticity similarly accounted for an indirect component of the adjusted association between narrative engagement and mental health awareness (*β* = 0.114, 95% bootstrap CI [0.058, 0.144]). The adjusted total standardized associations were *β* = 0.473 for narrative engagement with cultural resilience and *β* = 0.440 for narrative engagement with mental health awareness. These adjusted coefficients should be distinguished from the corresponding zero-order correlations, which were *r* = 0.51 and *r* = 0.44, respectively. The correlation pattern additionally indicated that greater mental health awareness was associated with lower mental health stigma and more favorable help-seeking attitudes. Diagnostic testing did not identify problematic multicollinearity, substantial heteroscedasticity or highly influential observations. Exploratory moderation analyses did not identify statistically significant interactions. Qualitative analysis complemented the quantitative findings by identifying cultural validation, normalization of psychological distress and intergenerational dialogue as prominent interpretive themes. Overall, the findings provide a multidimensional description of associations among documentary narrative engagement, cultural processes and mental-health-related constructs within the participating communities. Given the cross-sectional design, these results do not establish temporal ordering or causal effects.

## 4 Discussion

The mixed-methods study examined the associations between engagement with Indigenous documentary narratives, perceived cultural authenticity, cultural identification, cultural resilience, and mental health-related outcomes among Indigenous and community-associated adults in Yunnan and Hunan, China. Across 480 participants recruited from Kunming, Chuxiong, Dali, and Xiangxi, the findings showed consistent associations among narrative engagement, perceived authenticity, cultural identification, cultural resilience, mental health awareness, stigma, and help-seeking attitudes. Narrative engagement was positively associated with cultural resilience and mental health awareness and inversely associated with stigma, while qualitative accounts provided contextual explanations concerning cultural affirmation, normalization of distress, and intergenerational dialogue. The findings represent cross-sectional statistical associations and should not be interpreted as evidence that documentary exposure causes changes in psychological or cultural outcomes.

A first key finding was that narrative engagement was significantly positively correlated with cultural resilience (*r* = 0.51, *p* < 0.001) and remained significantly associated after adjustment for age, gender, education, residence, baseline cultural participation, and prior media exposure (*β* = 0.28, *p* < 0.001). Perceived authenticity demonstrated a similarly strong adjusted association with cultural resilience (*β* = 0.28, *p* < 0.001), and the model explained 53.4% of the variance (adjusted *R*^2^ = 0.526). The representation of participants across four recruitment sites and five reported ethnic/community categories provides contextual breadth to the sample. These findings are consistent with the possibility that representation quality may be relevant when audiovisual narratives address culturally significant domains including traditional knowledge, intergenerational transmission, community connections, and collective resilience (Maushe et al., 2025). Qualitative findings supported this interpretation, with participants describing documentary depictions as affirming heritage when cultural practices were portrayed as resources for living rather than inert or decorative traditions (Weems, 2025). The study did not establish temporal ordering, the observed associations cannot determine whether narrative engagement or perceived authenticity preceded differences in cultural resilience.

Cultural identification appeared to represent an important statistical indirect pathway. Narrative engagement was positively associated with cultural identification, while cultural identification was positively associated with cultural resilience. In the covariate-adjusted statistical indirect-association model, the association between narrative engagement and cultural identification was *β* = 0.512 (*p* < 0.001), the association between cultural identification and resilience was *β* = 0.440 (*p* < 0.001), and the adjusted direct association remained significant (*β* = 0.248, *p* = 0.002). The bootstrap indirect association was *β* = 0.225 (95% BCa CI [0.168, 0.291]; *p* < 0.001), with an adjusted total association of *β* = 0.473 (*p* < 0.001). This adjusted total association differs from the zero-order correlation (*r* = 0.51) because the former accounts for the prespecified covariates, whereas the latter represents the unadjusted bivariate association. This pattern is theoretically congruent with narrative and social-identity perspectives, in which culturally recognizable stories may supply symbolic content to which people assign meaning about group identity and continuity. The indirect pathway therefore provides evidence of a statistically significant indirect component in the observed association, rather than evidence of a temporally ordered or causal mediation process.

Another key finding was that mental health awareness was positively associated with narrative engagement (*r* = 0.44, *p* < 0.001) and remained independently associated after adjustment (*β* = 0.33, *p* < 0.001). Perceived authenticity also demonstrated an independent association with mental health awareness (*β* = 0.17, *p* < 0.001), while the model explained 34.8% of its variance (adjusted *R*^2^ = 0.337). The covariate-adjusted indirect association through perceived authenticity was *β* = 0.114 (95% CI [0.058, 0.144]; *p* < 0.001), with an adjusted total association of *β* = 0.440 (*p* < 0.001). These findings indicate that narrative engagement and perceived cultural authenticity were statistically associated with mental health awareness and that perceived authenticity accounted for an indirect component of the observed association. These findings are consistent with the possibility that culturally credible representation may be relevant to how audiences receive mental-health information embedded in documentary narratives (Sauda, 2025b). Participants described emotional distress using culturally familiar language and community-based frameworks, suggesting that psychological well-being was interpreted within rather than separately from cultural context.

Mental health awareness was positively related to help-seeking attitudes (*r* = 0.58, *p* < 0.001) and inversely related to stigma (*r* = −0.52, *p* < 0.001), while narrative engagement was inversely related to stigma (*r* = −0.35, *p* < 0.001). Accordingly, the previously stated interpretation that documentary engagement “directly reduces stigma and increases help-seeking behavior” has been revised: the present data show associations among narrative engagement, stigma, mental health awareness, and help-seeking attitudes, but do not establish that documentary engagement directly changes stigma or help-seeking behavior. Intergenerational dialogue emerged as an important qualitative context, with participants describing conversations between younger and older family members concerning historical adversity, cultural traditions, loss, coping, and emotional well-being (Cruz and Miranda, 2022). These accounts complement the quantitative associations by illustrating how culturally grounded narratives may provide a shared language for discussing identity and psychological experiences within families and communities. The qualitative findings therefore function as contextual interpretation of the quantitative patterns rather than as evidence of causal mechanisms.

Methodologically, the study benefited from *a priori* sample-size planning, explicit participant-flow documentation, culturally informed documentary selection, psychometric evaluation, CFA, multicollinearity and regression diagnostics, bootstrap estimation, and qualitative analysis. The final 60-item, six-factor CFA demonstrated acceptable model fit (*χ*^2^(1695) = 3284.15, *χ*^2^/df = 1.937, CFI = 0.941, TLI = 0.938, RMSEA = 0.044, 95% CI [0.042, 0.046], SRMR = 0.048), while reliability and convergent/discriminant validity indicators supported the measurement structure. The finalized measurement model comprised cultural identification, cultural resilience, narrative transportation, perceived cultural authenticity, mental health awareness, and mental health stigma. Community consultation, bilingual procedures, reflexive analysis, and review of translated quotations further strengthened cultural and interpretive credibility. Exploratory moderation analyses also found no statistically significant interactions across the tested terms (*p* > 0.35), including prior media exposure × narrative engagement for mental health awareness (*β* = 0.038, *p* = 0.412) and age × perceived cultural authenticity for resilience (*β* = −0.021, *p* = 0.631). Thus, the tested interaction terms did not provide statistical evidence that these contextual characteristics altered the magnitude of the observed associations. These null interaction findings should nevertheless be interpreted cautiously because the study was not primarily designed to detect small moderation effects.

The community-based purposive sampling strategy limits statistical generalizability beyond the participating regions and populations. Future studies should therefore use larger stratified or multilevel samples to examine community-specific patterns. Self-report measures may also be influenced by social desirability, particularly for culturally sensitive constructs including stigma and help-seeking. Documentary exposure was standardized, but the study did not establish long-term changes in attitudes or behavior. Moreover, the cross-sectional design prevents determination of temporal ordering and causal direction; Individuals with stronger cultural resilience or greater mental health awareness may also be more likely to engage deeply with Indigenous media, or the observed relationships may reflect reciprocal or unmeasured influences. The use of a single cross-sectional assessment also means that the statistical indirect-association findings should not be interpreted as evidence of causal pathways. In addition, the pooling of participants across four recruitment sites provides contextual breadth but does not establish that the observed associations are equivalent across communities or ethnic groups.

Future longitudinal and experimental studies should incorporate pre–post measurement, appropriate comparison conditions, repeated exposure assessments, behavioral or observed help-seeking outcomes, and culturally stratified or multilevel analyses. Such designs would allow temporal ordering and potential causal mechanisms to be evaluated more directly than was possible in the present study. The findings indicate that Indigenous documentary narratives are statistically embedded within a broader network connecting narrative engagement, perceived authenticity, cultural identification, cultural resilience, mental health awareness, stigma, and help-seeking attitudes. Within the limits of the cross-sectional design, the results support considering culturally grounded documentary media as a potentially meaningful context for identity-related and mental-health communication processes, rather than as an established causal intervention. Future community-partnered research should determine whether repeated exposure to culturally authentic narrative interventions is associated with sustained changes in cultural continuity, mental-health communication, stigma, and help-seeking behavior using longitudinal or experimental designs.

## 5 Conclusion

This study demonstrates that engagement with Indigenous documentary narratives is significantly associated with cultural resilience and mental health-related outcomes among Indigenous and community-associated adults in Yunnan and Hunan, China. Narrative engagement and perceived cultural authenticity showed independent positive associations with cultural resilience and mental health awareness, while cultural identification and perceived authenticity emerged as statistically significant indirect pathways linking narrative engagement with these outcomes. Higher narrative engagement was additionally associated with lower mental health stigma and more favorable help-seeking attitudes. The quantitative findings were complemented by qualitative accounts emphasizing cultural mirroring, normalization of emotional distress through culturally meaningful language and intergenerational dialogue as important dimensions of participants’ documentary experiences.

The findings highlight the importance of cultural authenticity, community participation and respectful representation when Indigenous narratives are used to communicate psychological well-being. Rather than presenting cultural heritage as a static or historical artifact, documentary narratives that reflect lived experiences, collective knowledge, family relationships and contemporary challenges may provide a culturally recognizable context for discussing resilience and mental health. Such approaches may be particularly valuable in communities where psychological experiences are communicated through culturally specific concepts and where formal mental-health terminology may not fully capture local understandings of distress and recovery.

Cross-sectional design cannot be used to make causal inferences. The consistency of psychometrically sound quantitative findings with the theoretically meaningful qualitative narratives of community-based researchers provides a strong interpretation of the observed associations and indirect statistical pathways and suggests that documentary exposure may lead to improvements in resilience, awareness, stigma and help-seeking, rather than proving that documentary exposure produces improvements in resilience, awareness, stigma, or help-seeking. Prospective evaluation using longitudinal and experimental designs, pre- and post-exposure assessments, sustained behavioral outcomes and evaluations of documentary interventions in multiple Indigenous communities should be conducted, while continuing to keep community governance at the center of documentary development, interpretation, dissemination and evaluation. The findings provide support for the potential value of culturally grounded documentary storytelling as a complementary communication approach for building cultural continuity and for building more culturally responsive conversations about mental health and highlight the importance of rigorous prospective evaluation prior to claims of intervention effectiveness.

## Data Availability

The original contributions presented in the study are included in the article/supplementary material, further inquiries can be directed to the corresponding author.
